# Angiogenesis and Its Therapeutic Opportunities

**DOI:** 10.1155/2013/127170

**Published:** 2013-07-28

**Authors:** So Young Yoo, Sang Mo Kwon

**Affiliations:** Laboratory for Vascular Medicine and Stem Cell Biology, Convergence Stem Cell Research Center, Medical Research Institute, Pusan National University School of Medicine, Yangsan 626-870, Republic of Korea

## Abstract

Angiogenesis plays critical roles in human physiology that range from reproduction and fetal growth to wound healing and tissue repair. The sophisticated multistep process is tightly regulated in a spatial and temporal manner by “on-off switch signals” between angiogenic factors, extracellular matrix components, and endothelial cells. Uncontrolled angiogenesis may lead to several angiogenic disorders, including vascular insufficiency (myocardial or critical limb ischemia) and vascular overgrowth (hemangiomas, vascularized tumors, and retinopathies). Thus, numerous therapeutic opportunities can be envisaged through the successful understanding and subsequent manipulation of angiogenesis. Here, we review the clinical implications of angiogenesis and discuss pro- and antiangiogenic agents that offer potential therapy for cancer and other angiogenic diseases.

## 1. Introduction

The growth of new capillaries from existing blood vessels, which is called angiogenesis, is mediated by a complex multistep process comprising a series of cellular events that lead to neovascularization [1, 2]. Angiogenesis plays a central role in various physiological processes within human body, not only during fetal development but also in tissue repair after surgery or trauma. Angiogenesis can be a hallmark of wound healing, the menstrual cycle, cancer, and various ischemic and inflammatory diseases [3–5]. The realization that tumor growth is associated with new blood vessels led us to investigate the chemical factors that mediate angiogenesis, broadened our knowledge of pathological processes, and thus opened new possibilities for the diagnosis and treatment of these diseases.

**Figure 1:**
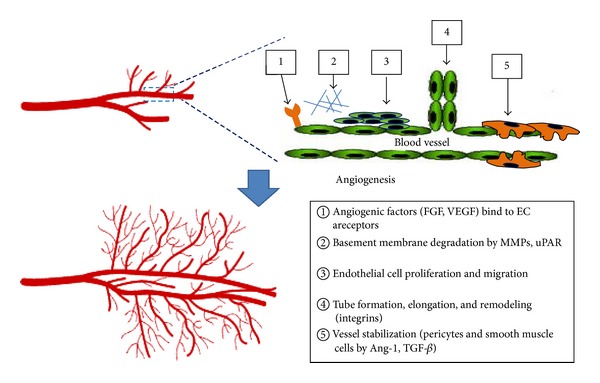
Processes in angiogenesis. (1) Angiogenic factors bind to their receptors on endothelial cells and activate the signal transduction pathways. (2) Matrix metalloproteinases are activated, and they degrade the extracellular matrix. (3) Endothelial cells migrate out of the preexisting capillary wall and proliferate. (4) Integrins are expressed by endothelial cells, facilitating their adhesion to the extracellular matrix and their migration for tube formation. (5) Angiopoietin 1 binds to Tie-2 receptors and stimulates pericyte recruitment and vessel stabilization.

The pivotal process of angiogenesis can be simply described as multiple steps. First, angiogenic stimuli cause increased endothelial cell (EC) permeability and cellular proliferation, which continues as the new capillary sprout elongates [6]. Second, proteolysis of basement membrane matricellular components is a necessary process to promote the invasion of ECs into the stroma of the neighboring tissue [7], in which the cooperative activity of the plasminogen activator (PA) system and matrix metalloproteinases (MMPs) is required. Third, migrated ECs trigger lumen formation as the sprout forms a multicellular structure. Then, a new capillary channel is formed. Finally, the capillary is stabilized through the construction of basement membrane, adherent junctions, and ECs (Figure 1).

A number of molecules are involved in these complex angiogenic cascades. Their names and functions are described briefly in the next section and are listed in Table 1. These factors are commonly used as the targets in strategies to manipulate angiogenesis. The pathological disruption of angiogenesis can be caused by either vascular insufficiency (myocardial or critical limb ischemia) or vascular overgrowth (hemangiomas, tumors, and retinopathies) (Table 2). Thus, therapeutic benefits may be realized by manipulating angiogenesis.

## 2. Angiogenic Factors and Regulation of Angiogenesis

In recent decades, numerous studies focused on identifying angiogenesis stimulators, which led to the identification of several angiogenic factors. Angiogenic factors can be categorized as follows: (1) soluble growth factors such as acidic and basic fibroblast growth factor (aFGF and bFGF) and vascular endothelial growth factor (VEGF), which are associated with EC growth and differentiation [8–10]; (2) inhibiting factors that inhibit the proliferation and enhance the differentiation of ECs, such as transforming growth factor *β* (TGF-*β*), angiogenin, and several low molecular weight substances [11–13]; and (3) extracellular matrix-bound cytokines that are released by proteolysis, which may contribute to the regulation of angiogenesis and include angiostatin, thrombospondin, and endostatin [14–18]. In addition, a number of microphages secreting bFGF, tumor necrosis factor (TNF), and VEGF were shown to be associated with tumor angiogenesis. Angiogenesis is governed by a balance between inducers and inhibitors [19]. It also can be regulated by EC proliferation, which is regulated or restrained by pericytes through the sequestration of potent mitogens in the extracellular matrix, changes in EC shape that reduce the sensitivity of the cells to growth factors, and certain endothelial integrins.

## 3. Angiogenic Disorders

The hypervascularity of tumors was first thought to be the inflammatory vasodilation of preexisting vessels in response to tumor metabolites and necrotic tumor products. It was also thought that tumor growth and metastasis depended on angiogenesis and that the tumor secreted the chemicals that shifted resting ECs into rapidly growing ECs. These ideas are not widely accepted yet. The concept of the role of angiogenesis in cancer and other diseases has now become clear.

### 3.1. Angiogenesis in Cancer

Most tumors (up to 2-3 mm^3^) persist *in situ* without neovascularization for months to years, but they become vascularized by “switches” in cells during the turnover to an angiogenic state when the tumor needs to keep growing. Cells in prevascular tumors may replicate as rapidly as those in expanding vascularized tumors; however, without the growth of new vessels, their growth is limited. A change in the local equilibrium between positive and negative angiogenic regulators of the growth of microvessels is involved in the switch to the angiogenic state [20, 21]. Of the angiogenic inducers (Table 1), those that are most commonly found in tumors appear to be VEGF and bFGF. Their angiogenic activities are synergistic [22]. VEGF plays a critical role in vasculogenesis and angiogenesis during fetal development. In a knockout mouse model, VEGF or VEGF receptor (VEGFR) inactivation resulted in defects in vasculogenesis in the early stages of development and was embryonic lethal [23].

VEGF is overexpressed in tumor stromal cells as well as tumor cells of renal cancer [24], lung cancer [25], breast cancer [26], and ovarian cancer [27]. VEGF expression is regulated positively by oncogenes such as Ras [28] and negatively by tumor suppressors such as von Hippel-Lindau (VHL) [29]. VEGF binds to VEGFR1 or VEGFR2 on the EC surface. Most of the angiogenic effects of VEGF result from VEGFR2 activation. Hypoxia is a major stimulator of VEGF expression that results from hypoxia-inducible factor 1 (HIF-1) binding to a hypoxia response element (HRE) within the VEGF promoter. It is also stimulated by inflammatory mediators (interleukin 1 (IL1), TGF-*β*, and prostaglandin E2 (PGE2)) or mechanical forces (shear stress and cell stretch). Increased PGE2 by cyclooxygenase-2 (COX-2) induces VEGF expression and angiogenesis in tumor cells or tumor stromal cells [30]. FGF signaling may be through the recruitment of other growth factor signaling pathways. The simultaneous VEGF signaling through VEGFR1 was required in the bFGF-stimulated capillary organization. bFGF also initiates the transcription of hepatocyte growth factor (HGF) [31]. However, the upregulation of an angiogenic inducer is not sufficient for tumor angiogenesis. The downregulation of certain negative regulators or inhibitors (Table 1) for vessel growth may be required [21].

Tumor growth is augmented by the onset of neovascularization through a perfusion effect and a paracrine effect. The perfusion effect is more efficient at allowing nutrients and oxygen to enter and catabolites to exit in crowded tissues, and the paracrine effect results from the production of growth factors (e.g., bFGF, insulin-like growth factor (IGF), platelet-derived growth factor (PDGF), and granulocyte colony stimulating factor (GCSF)) by ECs or their release by macrophages and other host cells that are delivered to the tumor by blood vessels. Neovascularization gradually reduces a tumor's accessibility to chemotherapeutic drugs because tumors compress their blood supply. Antiangiogenic therapy in rodents showed increased delivery of chemotherapy agents to a tumor [32], which may be associated with lowered interstitial pressure and an unpacked mass of tumor cells.

### 3.2. Angiogenesis in Other Diseases

Either excessive or deficient angiogenesis can be classified as an angiogenic disease, which is an abnormal growth of microvessels (Table 2). Recent studies reported that the angiogenic protein VEGF is the chief mediator of ocular neovascularization, a major cause of blindness worldwide [33, 34]. The upregulation of VEGF is a response to the local hypoxia that is produced in tumors by vascular compression and ischemia. Thus, a similar process may cause the formation of collateral vessels in an ischemic heart or limb. Neovascularization in atherosclerotic plaques [35] may be mediated by the overexpression of VEGF and by local hypoxia, which contribute to the growth and rupture of plaques. The expression of both VEGF and bFGF is excessive in hemangiomas of infancy. In the case of rheumatoid arthritis, infiltrated macrophages, immune cells, or inflammatory cells produce excessive angiogenic factors that may mediate the ingrowth of a vascular pannus in a joint [36]. In psoriasis, hypervascular skin lesions over expressed the angiogenic polypeptide IL8 and under expressed the angiogenic inhibitor thrombospondin 1 (TSP-1) [37]. Peptic ulcers in animals appear to be deficient in microvessels in the ulcer bed. The oral administration of aFGF induced angiogenesis in the ulcer bed and accelerated the healing of ulcers in animals [38]. Bowel atresia, vascular malformations, hemangiomas, and unilateral facial atrophy are developmental disorders that are caused also by abnormal vascular development through defects in angiogenesis [39].

## 4. Angiogenesis in Clinical Applications

Abnormal angiogenesis is the major cause of numerous diseases; therefore, angiogenesis itself can be useful for diagnostic/prognostic applications and can be manipulated for further clinical applications. In the case of ischemic diseases, which develop because of deficient angiogenesis, an angiogenesis stimulator can be used to induce therapeutic angiogenesis. In the case of cancer, which has excessive angiogenesis, angiogenic inhibitors including antiangiogenic factors can be used to attenuate angiogenesis. The Food and Drug Administration (FDA) approved becaplermin (Regranex, recombinant human PDGF-BB) for diabetic foot ulcer disease in 1977, which was the first angiogenesis-stimulating drug that was used as a therapeutic angiogenesis approach. For cancer therapeutics, the FDA approved bevacizumab (Avastin, humanized anti-VEGF monoclonal antibody), an angiogenesis inhibitor, to treat metastatic colorectal cancer in 2004 [40].

### 4.1. Diagnostic and Prognostic Applications

The quantitation of angiogenesis in a biopsy specimen may help predict the risk of metastasis or recurrence. The quantitation of microvessel density in histologic specimens of invasive breast cancer, for example, has provided an indication of the risk of metastasis [41]. A positive association between tumor angiogenesis and the risk of metastasis, tumor recurrence, or death has also been reported with regard to breast cancers and other types of tumors [42–44]. A high microvessel density may be a successful predictor of metastatic risk. An increased area of the vascular surface because of a high density may facilitate the escape of cancer cells into the circulation. An angiogenic cell that is shed from a primary tumor is more likely than a nonangiogenic cell to develop into a detectable metastasis.

The quantitation of angiogenic proteins in body fluids can be used to indirectly measure angiogenic activity. Higher concentrations of bFGF were found in the serum and urine of nearly 10% and more than 37% of cancer patients, respectively [45]. Concentrations of biologically active bFGF were abnormally high in the cerebrospinal fluid of children with brain tumors but not in children with hydrocephalus or malignant disease outside the central nervous system [46].

### 4.2. Therapeutic Angiogenesis

Therapeutic angiogenic drugs that accelerate the angiogenesis process are useful for treating diseases of deficient angiogenesis. In preclinical studies, orally administered bFGF was shown to stimulate angiogenesis and to accelerate the healing of duodenal ulcers in rats [38]. Phase I clinical trials were then prompted to evaluate this therapy in patients with gastric or duodenal ulcers that were refractory to conventional therapy [47]. Orally administrated bFGF was found to heal gastric ulcers that were caused by nonsteroidal anti-inflammatory drugs. Orally administrated aFGF induced angiogenesis in the ulcer bed and accelerated the healing of ulcers in animals [38].

A phase I clinical trial of therapeutic angiogenesis using bFGF protein in heparin-alginate slow-release microcapsules in 8 patients with symptomatic, severe coronary artery disease that was not amenable to complete revascularization by either percutaneous transluminal coronary angioplasty or coronary artery bypass grafting demonstrated the feasibility and safety of bFGF administration for coronary artery diseases [48]. These clinical studies suggest that angiogenic and other growth factors can heal gastrointestinal ulceration as well as coronary artery diseases. It is interesting to note that the antiulcer drug sucralfate (sucrose aluminum sulfate or Carafate) appears to act by protecting endogenous mucosal bFGF from degradation by acid.

Angiogenic agents can be used in ischemic heart disease as a therapeutic angiogenesis approach for cardiac tissue repair and regeneration [49, 50]. The therapeutic goal of this condition is to stimulate angiogenesis to improve perfusion, deliver survival factors to sites of tissue repair, mobilize regenerative stem cell populations, and ultimately restore form and function to the tissue. Although more than 2,000 patients with heart disease have received some form of experimental angiogenic therapy [51, 52], currently there are no FDA-approved angiogenic drugs to treat ischemic cardiovascular disease. The first FDA-approved device to stimulate new blood vessel growth in diseased hearts is a laser that is used in a technique called direct myocardial revascularization (DMR) or transmyocardial revascularization (TMR).

## 5. Antiangiogenic Therapies

Many studies using ECs that were isolated from either capillaries or large vessels led to considerable insights into the molecular and cellular biology of angiogenesis and the discovery and evaluation of potential antiangiogenic compounds (Table 3). They were identified using classical angiogenesis assays such as the chick chorioallantoic membrane (CAM), rabbit cornea assay, sponge implant models, and matrigel plugs [53–57]. Recent research has focused on the specific effects of antiangiogenic compounds on individual angiogenic processes. In this section, we discuss their clinical applications as antiangiogenic therapies.

### 5.1. Interferon Alpha-2a to Treat Hemangiomas

Hemangiomas occur in 1 out of 100 neonates and in 1 out of 5 premature infants [58]. Most do not need treatment because these tumors grow rapidly in the first year of life (the proliferating phase), slow down during the next 5 years (the involuting phase), and gradually regress by the age of 10–15 years (the involuted phase). However, about 10% of them may have serious tissue damage that includes interfering with a vital organ, obstructive airway, heart failure, or Kasabach-Merritt syndrome. Kasabach-Merritt syndrome, a platelet-trapping thrombocytopenic coagulopathy, and hepatic hemangiomas have a mortality rate of 30%–50%. Corticosteroid therapy worked for 30% of hemangiomas [59]. Radiation, cyclophosphamide treatment, and embolization were also tried and showed favorable outcomes; however, sometimes they showed toxicity. Interferon alpha-2a (IFN*α*-2a) is an angiogenic agent that could be useful for treating these hemangiomas. It was successfully used in a 7-year-old child with pulmonary hemangiomas [60]. It was also found that therapy with IFN*α*-2a accelerated the tumor regression in 18 of 20 hemangioma patients [61]. IFN*α*-2a suppresses the production of FGFs in human tumor cells, which could work for hemangiomas because bFGF is an angiogenic factor that is overexpressed in hemangiomas.

### 5.2. Ocular Neovascularization

Ophthalmology angiogenesis in the eye, an ocular neovascularization, includes age-related macular degeneration (AMD), proliferative diabetic retinopathy (PDR), diabetic macular edema (DME), neovascular glaucoma, corneal neovascularization (trachoma), and pterygium. Inhibiting VEGF is presently an antiangiogenic therapy that is approved for ophthalmic conditions. Two currently approved antiangiogenic therapies for ophthalmic diseases are an anti-VEGF aptamer (pegaptanib, Macugen) and a Fab fragment of a monoclonal antibody directed against VEGF (ranibizumab, Lucentis). A photodynamic therapy called Visudyne (QLT Therapeutics/CIBA Vision) has shown effectiveness for treating macular degeneration and was the first FDA-approved blood vessel therapy for eye disease in 2004 [62, 63].

### 5.3. Rheumatoid Arthritis

Clinical trials of angiogenic inhibitors have not been performed yet in patients with arthritis; however, minocycline and TNP-470 (also known as AGM-470) have shown efficacy as potent inhibitors of the vascular pannus in experimental arthritis [36, 64].

### 5.4. Cancer

Angiogenesis plays a critical role in the growth and spread of cancer because a blood supply is necessary for tumor growth and metastases. Tumors secrete chemical signals that stimulate angiogenesis and thus stimulate nearby normal cells. Therefore, many natural or synthetic angiogenesis inhibitors, also called antiangiogenic agents, have been studied to prevent or slow the growth of cancer. These inhibitors can interfere with angiogenesis in various ways. Bevacizumab (Avastin) is a monoclonal antibody that specifically recognizes and binds to VEGF, which prevents VEGF from activating VEGFR [40]. In contrast, other angiogenesis inhibitors, including sorafenib and sunitinib, bind to receptors on the ECs or to other proteins in the downstream signaling pathways to block their activities [65].

Bevacizumab was FDA-approved to be used alone to treat glioblastoma and to be used in combination with other drugs to treat metastatic colorectal cancer, some nonsmall cell lung cancers, and metastatic renal cell cancer. Bevacizumab is the first approved angiogenesis inhibitor that was shown to slow tumor growth and, more importantly, to extend the lives of patients with some cancers. The other FDA-approved antiangiogenic drugs are sorafenib (Nexavar) for hepatocellular carcinoma and kidney cancer, sunitinib (Sutent) for kidney cancer and neuroendocrine tumors, pazopanib (Votrient) for kidney cancer and neuroendocrine tumors, and everolimus (Afinitor) for kidney cancer. Researchers are exploring the use of angiogenesis inhibitors in some clinical trials (Table 4).

### 5.5. Conventional Anticancer Therapy versus Antiangiogenic Therapy

Conventional anticancer therapy generally faces the problems of drug resistance and impaired drug delivery because of genomic instability and the tumor mass of cancers [66]. Tumor mass has an interstitium composed of a collagen-rich matrix between cancer cells and vascular cells. Anticancer drugs need to pass through the interstitium from blood vessels to reach the cancer cells. However, tumor mass has an interstitium with an abnormally high interstitial fluid pressure, which acts as a barrier to drug delivery. However, angiogenesis inhibitors inhibit the growth of blood vessels rather than tumor cells. In some cancers, angiogenesis inhibitors are most effective when they are combined with additional therapies, especially chemotherapy. It has been hypothesized that these drugs help normalize the blood vessels that supply the tumor, facilitating the delivery of other anticancer agents [32]. Angiogenesis inhibitor therapy may prevent tumor growth instead of killing tumors. Therefore, antiangiogenic therapy may require a long period for treatment.

## 6. Concluding Remarks and Prospects

Currently, chemotherapeutic drugs are being used to treat cancer as well as other diseases. Unfortunately, many compounds showed limited efficacy with impaired delivery, penetration, and selectivity for the tumor cells, thereby causing serious side effects and bystander effects. The activity of these compounds is mainly restricted by the drug resistance of tumor cells. Preclinical studies and clinical trials suggest that angiogenesis-based therapy may be useful in the future care of patients. In particular, antiangiogenic therapy is a unique approach to kill tumor cells because it does not directly target cancer cells; instead, it inhibits the growth of blood vessels. So far, antiangiogenic agents are not likely to result in bone marrow suppression, gastrointestinal symptoms, or hair loss. The approach to slow the growth of blood vessels may require several months to a year; thus, the administration of the agents at lower doses and longer uninterrupted periods than the usual doses and periods of conventional cytotoxic agents should be considered in the design of clinical trials. The development of resistance to angiogenic inhibitors has not been a big problem so far. Furthermore, a combination of antiangiogenic therapy and conventional therapy may be more effective than either therapy alone. Angiogenesis-based therapy may provide a novel, selective, safe, and reasonable treatment in future medicine.

## Figures and Tables

**Table 1:** Overview of the different angiogenic factors.

Category	Names	Major functions	References
Proteolytic enzymes	(i) Matrix metalloproteinases (MMPs): matrilysin (MMP-7), interstitial collagenase (MMP-1), neutrophil collagenase (MMP-8), collagenase-3 (MMP-13), stromelysin-1 (MMP-3), stromelysin-2 (MMP-10), stromelysin-3 (MMP-11), metalloelastase (MMP-12), MMP-19, enamelysin (MMP-20), gelatinase A (MMP-2), gelatinase B (MMP-9), MT1-MMP (MMP-14), MT2-MMP (MMP-15), MT3-MMP (MMP-16), MT4-MMP (MMP-17) (ii) Plasminogen activators (PAs)	MMPs; taking different substrates according to MMPs; substrates can be collagen, gelatin, laminin, fibronectin, proteoglycans, and proMMPs	[67–69]

Angiogenesis inducers	Vascular endothelial growth factor family (VEGF-A or VEGF, P1GF, VGGF-B, VEGF-C, VEGF-D, orf virus VEGF or VEGF-E), fibroblast growth factor family (aFGF, bFGF, etc.), angiopoietin 1 (Ang-1), transforming growth factor-alpha/beta (TGF *α*/*β*), platelet-derived growth factor (PDGF), hepatocyte growth factor/scatter factor (HGF/SF), tumor necrosis factor-alpha (TNF*α*), interleukin-1/8, angiogenin, ephrins, integrins *α*_*v*_ *β*_3_, *α*_*v*_ *β*_5_, *α*_5_ *β*_1_, cyclooxygenase-2 (COX-2)	(i) Induction of EC proliferation, migration, and differentiation (ii) TGF-*β* shows opposite effect in some contexts	[8–10, 70–79]

Angiogenesis inhibitors	Thrombospondin-1/2 (TSP-1/2), angiostatin (plasminogen fragment), endostatin (collagen XVIII fragment), vasostatin (calreticulin fragment), tumstatin, platelet factor-4 (PF4), antiangiogenic antithrombin III, kringle 5 (plasminogen fragment), prolactin 16-kD fragment, fragment of SPARC, 2-methoxyestradiol, metalloproteinase inhibitors (TIMPs), interferon-alpha/beta/gamma (IFN *α*/*β*/*γ*), interleukin-12 (IL-12), IP-10, Ang-2	(i) Inhibit EC proliferation/migration (ii) Induce EC apoptosis (iii) TIMPs: inhibit MMP or uPA activity (iv) Ang-2: inhibit blood vessels maturation, antagonist of Ang-1	[11–15, 18, 80–85]

**Table 2:** Clinical manipulation of angiogenesis.

Therapeutic goal	Diseases	Definitions/symptoms	Reference
Inhibition of angiogenesis	Hemangiomas	Benign and usually a self-involuting tumor (swelling or growth) of the endothelial cells that line blood vessels and is characterised by increased number of normal or abnormal vessels filled with blood	[86]
	Psoriasis	Immune-mediated disease that affects the skin. The immune system mistakes a normal skin cell for a pathogen and sends out faulty signals that cause overproduction of new skin cells	[37]
	Kaposi's sarcoma	Tumor caused by human herpesvirus 8 (HHV8)	[87]
	Ocular neovascularization	Abnormal or excessive formation of blood vessels in the eye	[88]
	Rheumatoid arthritis	Inflammatory response of the capsule around the joints (synovium), secondary to swelling (hyperplasia) of synovial cells, excess synovial fluid, and the development of fibrous tissue (pannus) in the synovium	[36]
	Endometriosis	A gynecological medical condition in which cells from the lining of the uterus (endometrium) appear and flourish outside the uterine cavity, most commonly on the membrane which lines the abdominal cavity	[89]
	Atherosclerosis	Artery wall thickens caused largely by the accumulation of macrophage white blood cells and promoted by low-density lipoproteins (LDL, plasma proteins that carry cholesterol and triglycerides)	[35]
	Tumor growth and metastasis	Tumor-associated neovascularization is involved in tumor growth, invasion, and metastasis	[4]

Stimulation of angiogenesis	Induction of collateral vessel formation: Myocardial ischemia, Peripheral ischemia, Cerebral ischemia	After blood vessels blockage (occulsion), collateral vessels can be developed to improve blood supply to the area.	[90]
	Wound healing	Intricate process in which the skin (or another organ-tissue) repairs itself after injury. Angiogenesis occurs concurrently with fibroblast proliferation when endothelial cells migrate to the area of the wound	[91]
	Reconstructive surgery	Surgery to restore the form and function of the body	

**Table 3:** Antiangiogenic compounds and their mechanism of action (adapted from references [2, 92–102]).

Inhibiting angiogenic process	Antiangiogenic compounds	Mechanism of action
Inhibitors of ECM remodeling	Batimastat, Marimastat, AG3340, Neovastat, PEX, TIMP-1,2,3,4	MMP inhibitors, block endothelial and tumor cell invasion
	PAI-1,2, uPA Ab, uPAR Ab, Amiloride	uPA inhibitors, block ECM breakdown
	Minocycline, tetracyclines, cartilage-derived TIMP	Collagenase inhibitors, disrupt collagen synthesis and deposition

Inhibitors of adhesion molecules	*α*_*v*_ *β*_3_ Ab: LM609 and Vitaxin, RGD containing peptides, *α*_*v*_ *β*_5_ Ab	Block EC adhesion, induce EC apoptosis
	Benzodiazepine derivatives	Antagonist of *α*_*v*_ *β*_3_

Inhibitors of activated ECs	Endogenous inhibitors: endostatin, angiostatin, aaAT	Block EC proliferation, induce EC apoptosis, inhibit angiogenic switch
	IFN-*α*, IFN-*γ*, IL-12, nitric oxide synthase inhibitors, TSP-1	Block EC migration and/or proliferation
	TNP-470, Combretastatin A-4	Block EC proliferation
	Thalidomide	Inhibits angiogenesis in vivo
	Linomide	Inhibits EC migration

Inhibitors of angiogenic inducers or their receptors	IFN-*α*, PF-4, prolactin fragment	Inhibit bFGF, Inhibit bFGF-induced EC proliferation
	Suramin and analogues	Bind to various growth factors including bFGF, VEGF, PDGF, inhibit EC migration and proliferation
	PPS, distamycin A analogues, bFGF Ab, antisense-bFGF	Inhibit bFGF activity
	Protamine	Binds heparin, inhibits EC migration and proliferation
	SU5416, soluble Flt-1, dominant-negative Flk-1, VEGF receptor, ribozymes, VEGF Ab	Block VEGF activity
	Aspirin, NS-398	COX inhibitors
	6AT, 6A5BU, 7-DX	TP antagonists

Inhibitors of EC intracellular signaling	Genistein	Tyrosine kinase inhibitor, blocks uPA, EC migration and proliferation
	Lavendustin A	Selective inhibitor of protein tyrosine kinase
	Ang-2	Inhibits Tie-2

**Table 4:** Selected angiogenesis inhibitors in clinical trials.

Inhibiting target	Drug	Sponsor	Clinical trials/mechanism	References
Epidermal growth factor receptor (EGFR)	Gefitinib (Iressa)	AstraZeneca and Teva	FDA-approved in 2003 for NSCLC/effective in cancers with mutated and overactive EGFR	[103, 104]
	Lapatinib (Tykerb)	GSK	FDA-approved in 2007 for breast cancer/dual tyrosine kinase inhibitor which interrupts the HER2/neu and epidermal growth factor receptor (EGFR) pathways	[105]
	Erlotinib (Tarceva)	Genentech/OSI pharmaceuticals/Roche	FDA-approved in 2005/used to treat nonsmall cell lung cancer, pancreatic cancer, and several other types of cancer	[106]
	Canertinib (CI-1033)	Selleck Chemicals	Phase II/irreversible tyrosine-kinase inhibitor with activity against EGFR, HER-2, and ErbB-4	[107]

VEGFR	Vatalanib (PTK787 or PTK/ZK)	Bayer Schering and Novartis	Phase III/it inhibits all known VEGF receptors, as well as platelet-derived growth factor receptor-beta and c-kit, but is most selective for VEGFR-2	[108]

VEGFR-2	IMC-1C11	ImClone Systems	Phase I/chimerized monoclonal antibody	[109]

VEGFR-3	mF4-31C1	ImClone Systems	Phase I/rat monoclonal antibody to murine VEGFR-3, which potently antagonizes the binding of VEGF-C to VEGFR-3	[110]

Multiple growth factor receptors	Imatinib (Glivec)	Novartis	FDA-approved in 2001/competitive tyrosine-kinase inhibitor used in the treatment of multiple cancers, most notably Philadelphia chromosome-positive (Ph^+^) chronic myelogenous leukemia (CML)	[111]
	Sunitinib (Sutent)	Pfizer	FDA-approved in 2006 for renal cell carcinoma (RCC) and imatinib-resistant gastrointestinal stromal tumor (GIST)/the simultaneous inhibition of receptors for platelet-derived growth factor (PDGF-Rs) and vascular endothelial growth factor receptors (VEGFRs)	[112]
	Sorafenib (Nexavar)	Bayer and Onyx pharmaceuticals	FDA-approved in 2005/a small molecular inhibitor of several tyrosine protein kinases (VEGFR and PDGFR) and Raf kinases (more avidly C-Raf than B-Raf)	[113]
	Pazopanib (Votrient)	GlaxoSmithKline	FDA-approved in 2009 for advanced renal cancer/multitargeted receptor tyrosine kinase inhibitor of VEGFR-1, VEGFR-2, VEGFR-3, PDGFR-a/*β*, and c-kit	[114]

VEGF	Bevacizumab (Avastin)	Genentech/Roche	FDA-approved in 2004 for metastatic colorectal cancer/humanized anti-VEGF mAb, licensed to treat various cancers including colorectal, lung, breast (outside the USA), glioblastoma (USA only), kidney, and ovarian	[115, 116]

Integrin*α*_*V*_ *β*_3_	Vitaxin	Applied molecular evolution	Phase II as a treatment for colorectal cancer/humanized monoclonal antibody against the vascular integrin *α*_*V*_ *β*_3_	[117]
